# Flexibility of Gut Microbiota in Ageing Individuals during Dietary Fiber Long‐Chain Inulin Intake

**DOI:** 10.1002/mnfr.202000390

**Published:** 2021-01-25

**Authors:** Mensiena B. G. Kiewiet, Marlies E. Elderman, Sahar El Aidy, Johannes G. M. Burgerhof, Hester Visser, Elaine E. Vaughan, Marijke M. Faas, Paul de Vos

**Affiliations:** ^1^ Immunoendocrinology Division of Medical Biology Department of Pathology and Medical Biology University of Groningen University Medical Center Groningen Hanzeplein 1 Groningen 9700 RB The Netherlands; ^2^ Host‐microbe metabolic interactions Groningen Biomolecular and Biotechnology Institute (GBB) University of Groningen Nijenborgh 7 Groningen 9747 AG The Netherlands; ^3^ Department of Epidemiology University Medical Center Groningen University of Groningen Groningen 9713 GZ The Netherlands; ^4^ Sensus (Royal Cosun) Oosterlijke Havendijk 15 Roosendaal 4704 RA The Netherlands

**Keywords:** chicory long‐chain inulin, elderly, immunology, microbiota, SCFA

## Abstract

**Scope:**

During ageing, dysbiosis in the intestinal microbiota may occur and impact health. There is a paucity of studies on the effect of fiber on the elderly microbiota and the flexibility of the aged microbiota upon prebiotic intake. It is hypothesized that chicory long‐chain inulin consumption can change microbiota composition, microbial fermentation products, and immunity in the elderly.

**Methods and Results:**

A double‐blind, placebo‐controlled trial is performed in healthy individuals (55–80 years), in which microbiota composition is studied before, during, and after two months of chicory long‐chain inulin consumption. Fecal short chain fatty acid concentrations, T cell subsets, and antibody responses against a Hepatitis B (HB) vaccine are measured as well. Inulin consumption modified the microbiota composition, as measured by 16S rRNA sequencing. Participants consuming inulin have higher microbial diversity and a relatively higher abundance of the *Bifidobacterium* genus, as well as *Alistipes shahii*, *Anaerostipes hadrus*, and *Parabacteroides distasonis*. While the immune responses remain unchanged, the isobutyric acid levels, an undesired fermentation product, tend to be lower in the inulin group.

**Conclusions:**

Overall, it is shown that the gut microbiota composition is still sensitive to chicory long‐chain inulin induced changes in an ageing population, although this did not translate into an improved immune response to an HB vaccine.

## Graphical abstract

During ageing, a dysbiosis in the microbiota composition may occur and so far it is unknown whether this dysbiosis is sensitive for adjustment by prebiotics. In healthy seniors, consumption of long‐chain inulin from chicory roots (2‐months intake) changed the microbiota composition, although this did not translate into an improved immune response to a hepatitis B vaccine.

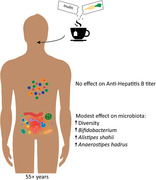

## 1 Introduction

During ageing, an undesired shift in the intestinal microbiota composition might occur, often referred to as dysbiosis.^[^ ^1^ ^]^ A shift towards a composition dominated by *Bacteroidetes* has been described in the elderly in multiple studies,^[^ ^2^ ^]^ as well as a gradual decrease in both the diversity and stability of the microbiota composition during ageing.^[^ ^3^ ^]^ In addition, health‐promoting species such as *Bifidobacterium* are reported to be decreased in the elderly intestine,^[^ ^4^, ^5^ ^]^ while at the same time the number of possibly harmful pathobionts increases with age.^[^ ^6^ ^]^ These changes in microbiota composition influence many ageing‐associated processes, such as a decrease in cognitive function, cardiovascular function, and immunosenescence.^[^ ^7^, ^8^, ^9^ ^]^ As immunosenescence results in increased susceptibility to pathogenic infections and lower responsiveness to vaccines, immunosenescence is considered to be a major contributor to morbidity and mortality in elderly.^[^ ^10^ ^]^

The human gut microbiome can be influenced by many factors,^[^ ^7^, ^11^ ^]^ and many studies have demonstrated the flexibility of the microbiome.^[^ ^12^ ^]^ This has created new opportunities for improving health by changing the microbiome. One promising way to modulate the microbiota composition is by consumption of non‐digestible dietary fibers.^[^ ^13^ ^]^ Inulin‐type fructans have been recognized by the International Scientific Association of Pro‐ and Prebiotics (ISAPP) as prebiotic, defined as “a substrate that is selectively utilized by the host microorganisms conferring a health benefit”.^[^ ^14^ ^]^ In addition to altering the abundance of, mainly bifidobacteria^[^ ^15^ ^]^ and some other species, the use of prebiotic inulin has also been shown to reduce inflammation,^[^ ^16^ ^]^ modify immune responses,^[^ ^17^, ^18^ ^]^ and improve bowel function^[^ ^19^, ^20^ ^]^ and blood markers such as insulin, glucose, and lipids.^[^ ^21^ ^]^ The microbiota changes are implicated in these benefits. More recently we have shown for example that long‐chain inulin was able to boost the immune response in healthy adolescents and induced an enhanced efficacy of Hepatitis B (HB) vaccination.^[^ ^22^ ^]^

Microbiota transfer studies in mice have confirmed a causal relationship between age‐related changes in microbiota and immunosenescent features.^[^ ^23^, ^24^ ^]^ The changed microbiota composition in the elderly may result in a modified production of microbial metabolites, including lipopolysaccharides and short‐chain fatty acids (SCFA).^[^ ^25^ ^]^ These SCFA play a key role in the effect of intestinal microbiota on health,^[^ ^25^ ^]^ since they are known for their ability to regulate immune functions. Butyrate, for example, is used for energy by the colonic intestinal cells and can modulate immunity.^[^ ^26^, ^27^ ^]^

However, as both the microbiota composition and immunity changes during ageing,^[^ ^3^, ^10^ ^]^ it is unknown whether inulin can modify the microbiota in a more elderly population. Much debate is going on about the reversibility of the microbiota composition in the elderly.^[^ ^28^, ^29^ ^]^ Therefore, the aim of this study was to determine the flexibility of the aged gut microbiota by investigating the impact of chicory long‐chain inulin consumption on microbiota composition using community‐wide 16S rRNA sequencing and production of microbiota fermentation products in the elderly. Additionally, we tested whether inulin could boost the immune system in these persons, which deteriorates during immunosenescence. To this end, a double‐blind placebo‐controlled trial in healthy individuals of 55 years or older was performed, in which we studied the long term impact (until 22 weeks after termination of inulin consumption) of a 2‐months period of chicory long‐chain inulin consumption on the microbiota composition and SCFA concentrations. To study whether the inulin intervention can change functional responses against a pathogen, T helper (Th) cell and memory T cell subsets and the antigen‐specific antibody response were analyzed while participants started a HB vaccine protocol (three vaccinations during 6 months) during the 2‐month inulin treatment period.

## 2 Experimental Section

### 2.1 Subjects and Inclusion Criteria

A double‐blind placebo‐controlled human dietary intervention trial was performed to study the effects of chicory long‐chain inulin on the microbiota composition and on immune responses to a vaccine in middle‐aged to elderly. All included participants were healthy, Caucasian individuals with an age between 55–80 years. Individuals with acute or chronic illness (e.g., diabetes mellitus), gastrointestinal disorders (e.g., inflammatory bowel disease), treatment with antibiotics within 6 months of the start of the study, prior HB vaccination or infection, or an immunodeficiency disorder were not included in the study. After inclusion participants were randomly allocated by an independent researcher to the inulin group or the control group (glucose) (n = 15 and n = 13, respectively). To check the compliance, the number of remaining supplement sachets was checked and two persons in the inulin group did not comply with the daily intake. Consequently, 13 subjects were available for both groups. During the study, two participants in the inulin group were treated with antibiotics between days 35 and 63 of the study due to bronchitis and cystitis. As a consequence, all data of these two participants were excluded from day 63 onwards. Thus, data of 13 subjects were available for the inulin group until day 63, while after day 63, data of 11 subjects were available for this group.

### 2.2 Study Design

**Figure 1:**
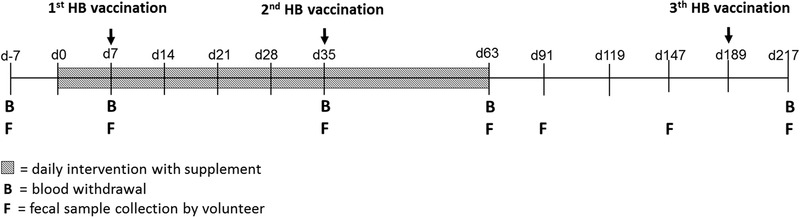
Experimental setup. Participants consumed either 8 g day^−1^ of long‐chain inulin or 5 g day^−1^ of placebo from day 0 till day 63 of the study (grey area). In order to study the microbiota composition, fecal samples (F) were collected on days −7, 7, 35, 63, 91, 147, and 217. To study the effects of inulin on immunity, the supplement intake period corresponded with the first 2 months of a HB vaccination program. Participants were given the first vaccine on day 7 of the study, followed by vaccinations on day 35 and day 189. On days −7, 7, 35, 63, and 217, venous blood (B) was collected from the participants to measure the anti‐HB titer and to analyze immune cell populations.

An overview of the experimental outline is shown in **Figure** **1**. Participants consumed either long‐chain inulin or placebo daily from day 0 till day 63 of the study. In order to study the microbiota composition and intestinal SCFA concentrations, participants collected and immediately froze (at −20 °C) fecal samples at home on days −7, 7, 35, 63, 91, 147, and 217. Samples were collected by the researchers on a regular basis and transported to the laboratory on dry ice and stored at −80 °C until further analysis.

To study the effects of inulin on immunity, the supplement intake period corresponded with the first 2 months of a HB vaccination program. HB vaccinations (Engerix, GlaxoSmithKline, Brentford, UK) were provided intramuscularly by a qualified physician (Premeo, Hoofddorp, the Netherlands) according to the standard Dutch vaccination scheme (0‐1‐6 months). Participants were given the first vaccine on day 7 of the study, followed by vaccinations on day 35 and day 189. On days −7, 7, 35, 63, and 217, venous blood was collected from the participants in order to measure the anti‐HB titer in the serum. This blood was also used to analyze multiple immune cell populations.

This study was approved by the ethical board of the University Medical Centre Groningen, documented as METc 2016/650. Written consent was obtained from all participants, and data were presented anonymously. All clinical investigations were conducted according to the principles of the declaration of Helsinki.

### 2.3 Dosage Information

**Figure 2:**
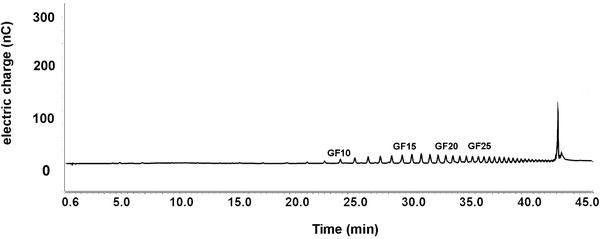
DP profile of long‐chain inulin obtained by HPAEC. Individual peaks indicate fructan oligomers. The chicory root fructans in this mixture are terminated by a glucose molecule, as indicated by GF. The following number indicates the number of fructose subunits, which corresponds with the DP. The DP in this inulin mixture ranges from 10 to 60.

The inulin used in the study (FrutafitTEX!, Sensus, Roosendaal, the Netherlands) was manufactured from native chicory inulin, and characterized by a degree of polymerization (DP) of 10–60, which makes it a long‐chain inulin. The DP profile obtained by High Performance Anion Exchange Chromotography (HPAEC) of the inulin is shown in **Figure** **2**. Glucose (Sigma Aldrich, Zwijndrecht, the Netherlands) was used as a placebo. Participants consumed either 8 g day^−1^ of long‐chain inulin or 5 g day^−1^ placebo daily from day 0 till day 63 of the study (8g inulin and 5g have the same visual volume). Participants were provided with identical white sachets containing inulin or placebo for the complete trial, and they were given instructions on how to consume the supplements. Participants were asked to dissolve the complete content of a sachet in a cup of tea, in order to ensure complete intake of the sachet. The exact time point of the day at which the supplement was taken was not fixed. The dosage of 8 grams of inulin per day has already been generally accepted as a functional dose for a long time, in terms of bifidogenic effects^[^ ^30^, ^31^ ^]^ as well as immune‐stimulatory effects.^[^ ^22^, ^32^ ^]^ Supplementation of 8 grams of inulin per day was also the best choice when considering the gastrointestinal tolerance of chicory inulin, since consumption of inulin products containing more than 10 grams per day results in GI symptoms like flatulence and bloating in healthy individuals.^[^ ^33^ ^]^

### 2.4 Estimation of Fiber Consumption from Regular Diet

During the study, the participants continued their normal diet. To monitor the daily fiber intake of the participants through their regular diet during the study, the participants filled out a questionnaire on day 0 and 63. In this questionnaire, they were asked to score how frequently (0 = never, or less than once a week, 1 = once a week, 2 = 2 to 3 times a week, 3 = 4 to 6 times a week, 4 = once a day or more) they consume twelve general fiber‐rich products (e.g., cereals, legumes, nuts, whole meal products). The sum of these scores was used as an estimation of the fiber intake of the participants through their regular diet during the course of the study. The averages of the individual scores of day 0 and 63 were used to compare the estimated fiber intake through their regular diet between the experimental groups. The same method was used to estimate the level of exercise.

### 2.5 Microbiota Analysis and Bioinformatics

Next‐generation 16S rRNA sequencing was used to classify and characterize the microbial communities in 182 collected stool samples. The complete process, including DNA isolation, purification, hypervariable region amplification by PCR, and sequencing were performed by GATC Biotech AG (Constance, Germany), together with the required quality controls (for more details see Supporting Information). Using microbial Ecology (QIIME) software, preliminary quality control steps were performed as described previously,^[^ ^34^ ^]^ and chimera sequences were removed with ChimeraSlayer. The remaining effective sequences were binned into operational taxonomic units with a cut‐off of 97% identity to determine alpha diversity (Shannon).

### 2.6 SCFA Analysis

The concentrations of acetic acid, propionic acid, isobutyric acid, butyric acid, isovaleric acid, and valeric acid in the collected fecal samples were analyzed by gas chromatography (GC) (for more details see Supporting Information).

### 2.7 Lymphocyte Staining and Flow Cytometry

Venous blood from the participants was collected in lithium heparin Vacutainer tubes (BD, Plymouth, UK). The blood was incubated for 10 min with ice cold ammonium chloride twice to eliminate red blood cells. After washing with ice cold FACS buffer (PBS + 10% dFCS (v/v)), the suspensions were resuspended in RPMI medium + 10% dFCS and counted using a coulter counter (Beckton Dickinson BV, Breda, the Netherlands).

White blood cells were stained for several T cell populations on day −7, 7, 35, 63, and 217. T cells were determined using CD3, and further subdivided into T cytotoxic (CD8+) and Th (CD4+) cells. CD45RO was used to select memory T cells. Within the Th cell population, Th1 (Tbet+) and T regulatory (Treg) cells (FoxP3+) were identified. The antibodies which were used are described in Table S1, Supporting Information. Details of the staining protocol are provided in the Supporting Information.

### 2.8 Anti‐HB Titer Analysis

Anti‐HB soluble antigen (HBsAG) titers were analyzed in the serum of the participants collected on day −7, 7, 35, 63, and 217 and stored at −80 °C. The Architect anti‐HBs assay, which is a chemiluminescent microparticle immunoassay, was performed to obtain the quantitative determination of the antibody to HBsAG in the samples using the Architect i2000 Analyzer (Abbott, Illinois, USA). The assay was performed following the manufacturer's instructions.

The effect of inulin intake on the number of vaccine responders was investigated as well. Participants were considered to be a responder when their titer was equal to or over 10 IU mL^−1^.^[^ ^35^ ^]^

### 2.9 Statistical Analysis

Graphpad Prism 5.0 (La Jolla, CA, USA) was used for graphical representation of the data. Statistical analyses were performed in SPSS (version 23, IBM, Armonk, NY, USA). To describe normally distributed data, mean and standard deviation were used, data with skew distribution were shown as the median and interquartile range (IQR). Independent data were compared with t‐tests or Mann–Whitney tests, depending on the distributions. Redundancy analysis (RDA) was used to investigate the difference in the global gut microbiota composition between the two groups. Repeated measures on outcome variables were used to calculate Area under Curves (AUC). Linear regression models (LRM) were used to test the effect of the treatment, correcting for baseline measures, on AUC. In case the distribution of an AUC was skew (to the right), the AUC was log‐transformed. A P‐value ≤0.05 was considered to be significant. A statistical trend was defined as 0.05 < *P* ≤ 0.1.

## 3 Results

### 3.1 Groups Showed no Significantly Different Characteristics

**Table** **1** shows the characteristics of the experimental groups. Both groups contained data of 13 subjects. Each group consisted of 9 males and 4 females. No significant differences were observed between the experimental groups in terms of the possible confounding factors age, Body Mass Index (BMI), and exercise score. In addition, the estimated fiber intake through the regular diet of the participants did not differ between the two groups.

**Table 1:** Participant characteristics

	Inulin	Placebo	*p*‐value^a)^
Number	13	13	
Sex [male:female]	9:4	9:4	
Age [mean years, SD]	62.2±6.9	63.7±8.1	0.64
BMI [mean kg m^−2^,SD]	26.1±3.6	29.7±5.6	0.06
Estimated exercise [mean score^b)^, SD]	2.5±0.9	2.4±1.2	0.85
Estimated dietary fiber intake [mean score^b)^, SD]	20.1±4.5	21.1±4.8	0.40

^a)^ Inulin and placebo group were compared using t‐tests

^b)^ Scores as based on a questionnaire explained in the Experimental Section

### 3.2 Fecal Microbiota Composition Changed by Inulin Consumption

**Figure 3:**
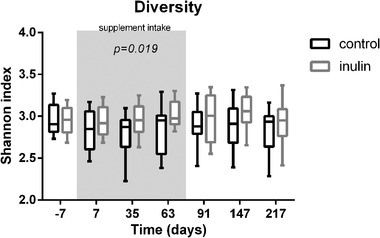
Shannon diversity of the fecal microbiota of subjects. Results are shown in box and whiskers plots. Boxes representing IQR and whiskers show the minimum and maximum. Using an LRM on the AUC, inulin treatment was found to increase diversity during the supplementation period (day 0 till day 63, grey area) (*p* = 0.019) and tended to maintain this increase till day 217 (*p* = 0.061).

It is well established that inulin is degraded by microbiota in the colon, which can lead to changes in microbiota composition, notably a bifidogenic effect.^[^ ^36^ ^]^ Therefore, we studied the microbiota composition and diversity in the stool of the participants throughout the study. In order to investigate the diversity of the microbiota composition at species level, the effects of inulin intake (treatment) on Shannon's index were analyzed. Subjects consuming inulin were found to have a significantly higher diversity (LRM, *p* = 0.019) as compared to the placebo consuming subjects during the supplement intake period. This increase tended to be maintained till day 217 (*p* = 0.061) (**Figure** **3**).

**Figure 4:**
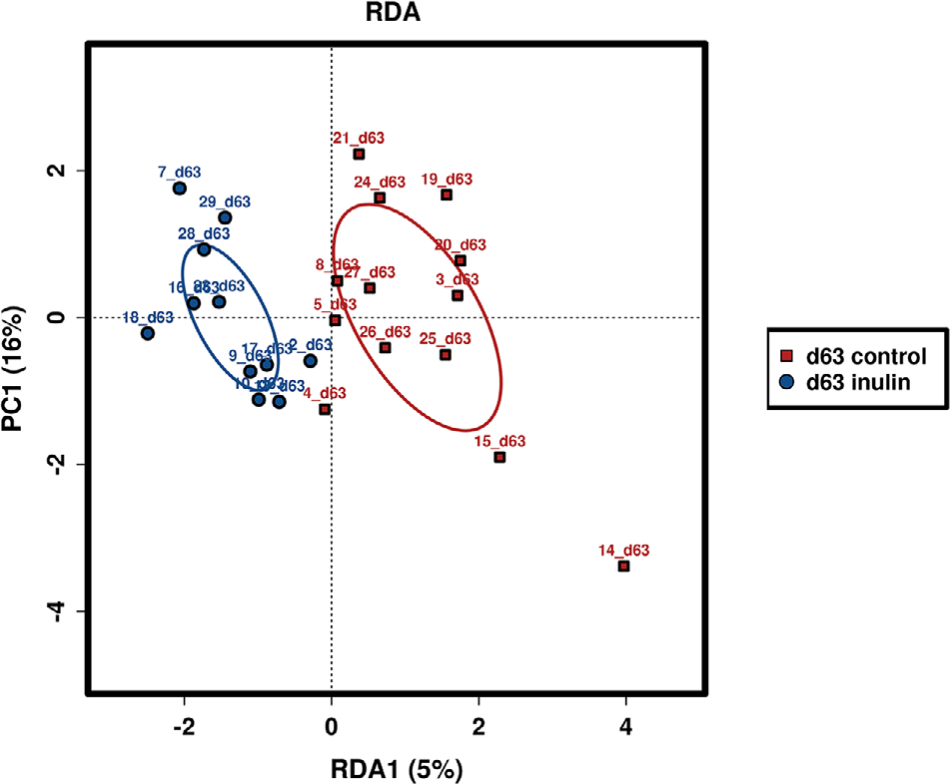
RDA plots showing the variation in global microbiota composition. No significant difference between the inulin and control groups was observed.

The difference in the global microbiota composition of the inulin and control group was explored by ordination. Statistics based on random permutations of the RDA showed no significant difference between the groups on the timepoint directly after the inulin intake period, day 63 (**Figure** **4**, *p* = 0.325). When focusing on the relative abundance of the phyla, we found that Firmicutes were the most abundant (65.9–73.2%), followed by Actinobacteria (21.3–25.9%), and Bacteroides (3.17–9.54%) in the feces of the volunteers. No significant differences between participants treated with inulin and placebo were observed in the relative abundance of these phyla during the supplement intake period (Figure S2, Supporting Information).

**Figure 5:**
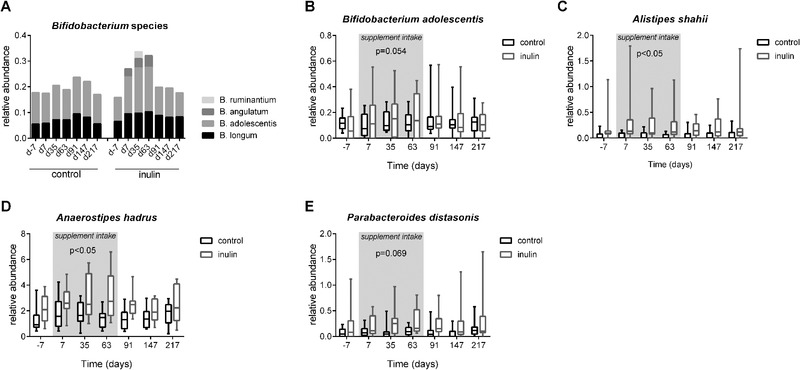
Relative abundance of A) *Bifidobacterium* species, B) *B. adolescentis*, C) *A. shahii*, D) *A. hadrus*, and E) *P. distasonis* detected in fecal samples. Results are shown in box and whiskers plots. Boxes representing IQR and whiskers show the minimum and maximum. Using an LRM on the AUC, inulin treatment was found to increase the relative abundance of *A. shahii* and *A. hadrus* during the supplementation period (day 0 till day 63, grey area) (both *p *< 0.05) and this increase was maintained till day 217 (*p *< *0*.05).

Next, we investigated the effect of treatment on the relative abundance of some individual bacteria species. Since inulin‐type fibers are known for their bifidogenic effects, changes in *Bifidobacterium* were investigated. Only during the supplement intake period, the presence of two extra *Bifidobacterium* species, *Bifidobacterium angulatum* and *Bifidobacterium ruminantium*, was detected in the inulin group and not in the control group (**Figure** **5**A). Furthermore, *Bifidobacterium adolescentis*, which was present in both groups at all‐time points, tended to have a higher (LRM, *p* = 0.054) abundance in the inulin group compared to the control group during the supplement intake period (Figure 5B). After termination of the inulin supplement, these changes were no longer detected after 4 weeks. The relative abundances of *Alistipes shahii* (Figure 5C) and *Anaerostipes hadrus* (Figure 5D) were significantly higher in the inulin group as compared to the control group during the supplement intake period (LRM, *p *< 0.05). The effect of inulin intake on both *A. shahii and A. hadrus* was maintained till week 22 after the termination of the inulin supplement (*p *< 0.05). The relative abundance of *Parabacteroides distasonis* (Figure 5E) tended to be higher (LRM, *p* = 0.069) in the inulin group. A complete list of the relative abundance of all detected phyla, family, genus, and species per individual is shown in Table S2, Supporting Information, while the mean relative abundances of the inulin and treatment groups, with the corresponding fold changes, P‐values and FDRs, have been provided for all analyzed taxonomical ranks and all time points in Table S3, Supporting Information.

### 3.3 Short‐Chain Fatty Acids not Changed after Inulin Consumption

**Figure 6:**
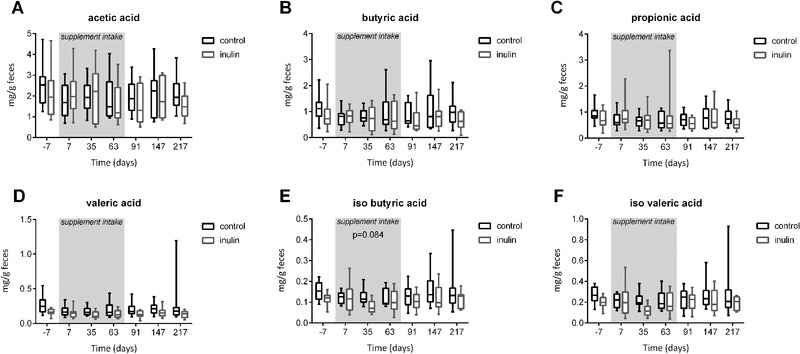
Concentrations of A) acetic acid, B) butyric acid, C) propionic acid, D) valeric acid, E) isobutyric acid, and F) isovaleric acid in fecal samples are shown in box and whiskers plots. Boxes representing IQR and whiskers show the minimum and maximum. Using an LRM on the AUC, no effect of inulin treatment was found during the supplementation period (day 0 till day 63, grey area).

Changes in diet or microbiota composition can cause differences in the composition of metabolites they produce. SCFAs are produced by gut bacteria during the digestion of non‐digestible polysaccharides, and can affect immune functioning.^[^ ^37^ ^]^ We analyzed the concentrations of acetic acid, propionic acid, isobutyric acid, butyric acid, isolvaleric acid, and valeric acid in the stool of the subjects during the supplement intake period (**Figure** **6**). A LRM showed that subjects consuming inulin tended to have a significantly lower (LRM, *p* = 0.084) amount of the branched SCFA isobutyric acid (Figure 6E), an undesired product of colonic proteolytic fermentation, as compared to subjects consuming the placebo.

### 3.4 Immune Parameters Unchanged by Inulin Intake with HB Vaccine

**Figure 7:**
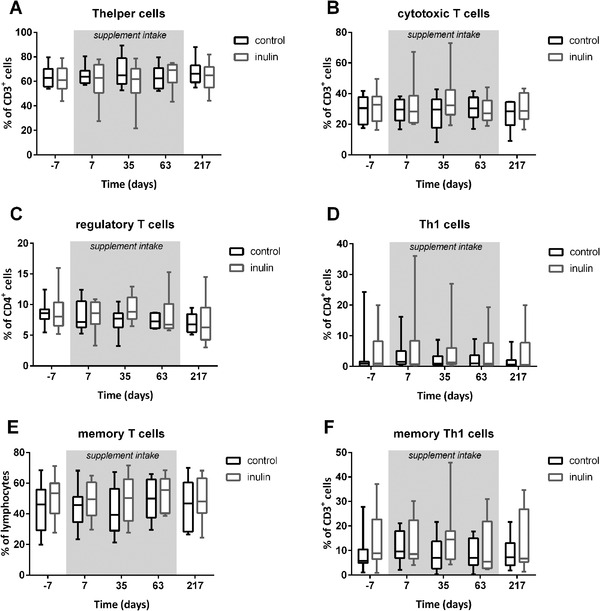
Percentages of A) Th cells, B) cytotoxic T cells, C) regulatory T cells, D) Th1 cells, E) total memory T cells, and F) memory Th1 cells in the blood. Results are shown in box and whiskers plots. Boxes representing IQR and whiskers show the minimum and maximum. Using an LRM on the AUC, no effect of inulin treatment was found during the supplementation period (day 0 till day 63, grey area).

To investigate the immune effects of inulin consumption on a cellular level, we investigated the effects of inulin intake on multiple T cell subpopulations on days 7, 35, 63, and 217. No effects of inulin consumption on the percentages of CD4+ and CD8+ have been observed (**Figure** **7**A,B). Furthermore, the intake of inulin did not have an effect on the CD4+/CD8+ ratio (results not shown), which is crucial for optimal immune functioning.^[^ ^38^, ^39^, ^40^ ^]^ The overall percentages of Treg and Th1 cells within the Th cell population were also not affected by inulin consumption (Figure 7C,D).

Vaccination leads to the production of memory T cells, which facilitate long‐term protection against the specific antigen.^[^ ^41^ ^]^ Therefore, the effect of inulin consumption on changes in the percentage of memory T cells was also investigated (Figure 7E,F). However, no effect of inulin on the percentage of memory T cells was found.

### 3.5 Anti‐HB Titers not Changed by Inulin Consumption

**Figure 8:**
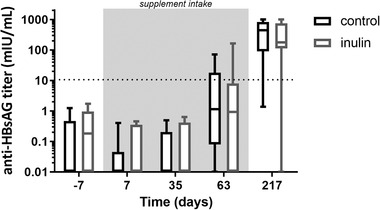
Anti‐HB antibody titers of the placebo and inulin groups over time. HB vaccination was given to all participants on day 0, 35, and 63 of the study. Results are shown in box and whiskers plots. Boxes representing IQR and whiskers show the minimum and maximum. Using an LRM on the AUC, no effect of inulin treatment was found during the supplementation period (day 0 till day 63, grey area).

Finally, we investigated whether inulin intake can change a functional immune response against a pathogen, by measuring antibody titers after a HB vaccination protocol. The anti‐HB antibody titer increased significantly over time in both groups (**Figure** **8**), which suggests that the vaccination program was effective in most subjects. However, inulin supplementation did not have a significant effect on the anti‐HB antibody titer levels (Figure 8).

Besides the analysis of quantitative anti‐HB antibody levels, the number of responders on day 63 and 217 was also assessed. A month after receiving the first 2 vaccinations (day 63), 3 responders were present in the placebo group, while 1 responder was present in the inulin group. In addition, a month after the third vaccination (final time point), 11 and 10 subjects were classified as responders in the placebo and inulin group, respectively. No significant effect of inulin supplementation on the frequency of responders was found (**Table** **2**).

**Table 2:** Responders and non‐responders to the HB vaccination

Timepoint	Inulin	Placebo	*p*‐value^a)^
D63	1 (9.1%)	4 (30.8%)	0.33
D217	10 (90.9%)	11 (84.6%)	1.00

^a)^ Inulin and placebo group were compared using Fisher's exact test.

## 4 Discussion

Ageing is related to a dysbiosis in the intestinal microbiota, which is associated with negative effects on general health.^[^ ^7^, ^8^, ^9^ ^]^ It is still largely unknown whether these ageing related changes in microbiota composition are reversible and manageable by prebiotics such as inulin. In this study, we show that the microbiota of an ageing population is still responsive to chicory long‐chain inulin fiber induced changes and adapts upon consumption of this inulin. These changes were not associated with changes in systemic immune populations after HB vaccination. This is the first study using community‐wide 16S rRNA sequencing to study the impact of specifically long‐chain inulin on the fecal microbiota, and notably in older subjects.^[^ ^42^ ^]^

There is evidence that the intake of a soluble fiber will induce a higher microbial diversity.^[^ ^43^ ^]^ In this study, the participants consuming the inulin also developed a higher microbial diversity than the participants consuming the placebo. This finding is, however, in contrast with other studies finding no effect of inulin on microbiota diversity.^[^ ^44^, ^45^ ^]^ Variable outcomes on alpha‐diversity after inulin consumption have indeed been recognized in a recent literature review.^[^ ^42^ ^]^ There may be different reasons for such differences such as the shorter supplementation period and younger age of the participants or health status (overweight) in these studies. Additionally, the effects may depend on the specific inulin structure, since native inulin and a FOS/inulin mixture were used in these studies. For example, inulin with a shorter DP may be fermented by bacteria before reaching the transcending and distal parts of the colon, and therefore may have different effects on the gut microbiota than longer DP inulin.^[^ ^46^ ^]^

The observed bifidogenic effect of inulin is in line with a previous study showing an overall increase of *Bifidobacterium* after the intake of 8 g day^−1^ of a chicory short‐ and long‐chain inulin mixture.^[^ ^31^ ^]^ We showed that only *B. adolescentis* and *B. longum* were detected before inulin intake was started. This corresponds with another study which showed that the total number of *Bifidobacterium* species decreases with age, but that *B. adolescentis* and *B. longum* remain present during ageing.^[^ ^47^ ^]^ Interestingly, during inulin consumption, two additional *Bifidobacterium* species, namely *B. angulatum* and *B. ruminantium*, appeared in the inulin group. *B. angulatum* has been described in in vitro studies as a potent and structure unspecific carbohydrate fermenter,^[^ ^48^, ^49^ ^]^ which explains its presence during the inulin consumption period. The delayed increase of *B. ruminantium* is more likely to be due to cross‐feeding (i.e., it lives off the products of another species), since other studies showed its inability to degrade inulin,^[^ ^50^ ^]^ due to a lack of glycosyl hydrolase genes.^[^ ^51^ ^]^

Initial research using culturing and targeted 16S rRNA techniques like fluorescent in situ hybridization showed the bifidogenic properties of inulin. However, sequencing of the 16S rDNA gene also allows the detection of potential effects of inulin on other species. Therefore, we performed a broader screening, and also found effects of inulin consumption on other species. Three species, *A. shahii*, *A. hadrus*, and *P. distasonis*, were particularly interesting. The timing of the increased relative abundance of these in the inulin group suggests that the increase is due to inulin intake. All three species have been shown to be beneficial in the intestine in terms of promoting health. A positive correlation between the number of *A. shahii* and species richness was previously found,^[^ ^52^ ^]^ which is generally associated with a healthier and more stable microbiota composition.^[^ ^53^ ^]^ Furthermore, both *A. shahii* and *P. distasonis* have been shown to modulate the immune system. Both species induced anti‐inflammatory effects such as an increase in Tregs in a colon cancer mouse model.^[^ ^54^ ^]^ On the other hand, *A. shahii* has also been described to boost the innate immune response during cancer immunotherapy in mice.^[^ ^55^ ^]^ Both intestinal health and anti‐inflammatory effects might be a result of their metabolic products, as both A*. shahii* and *P. distasonis* have been suggested to produce butyrate.^[^ ^56^, ^57^ ^]^ *A. hadrus* is among the most abundant butyrate producers in the human intestine, and therefore also plays a prominent role in overall health.^[^ ^58^ ^]^ Recently, Baxter et al. also detected an increased relative abundance of *A. hadrus* after supplementing the diets of healthy young adults with 20g of chicory native inulin.^[^ ^59^ ^]^

Despite a higher abundance of acetate and butyrate‐producing bacterial species, no overall effect of inulin intake was observed on the concentration of butyric acid, acetic acid, and propionic acid in the feces. Although inulin has been shown to robustly produce SCFA in numerous in vitro gut models.^[^ ^60^ ^]^ However, data from human studies vary. Some studies showed the expected higher levels of SCFAs in the stool of inulin consuming individuals,^[^ ^61^ ^]^ other similar studies found no effects,^[^ ^44^ ^]^ or even described a decrease.^[^ ^62^ ^]^ This absence of enhanced SCFA levels in the stool does not necessarily mean that the long‐chain inulin did not stimulate SCFA production in the intestine. There might be several explanations. One explanation is that the dose of inulin used might be too low to obtain measurable effects in feces since most significant increases in SCFA concentrations in stool and serum have been found shortly after intake of a high amount of inulin (15–24 g).^[^ ^61^, ^63^ ^]^ Another, more likely, explanation based on our experience in animal studies, is that SCFAs levels were enhanced at the primary site of production, that is, in the cecum or other parts of the colon,^[^ ^64^, ^65^, ^66^ ^]^ but not in the stool due to being rapidly utilized by the colonocytes for energy, consumed in bacterial cross‐feeding and absorbed into the host circulation. A stable‐isotope dilution method using intravenous infusion with ^13^C‐SCFAs confirmed the in vivo colonic production of SCFAs in healthy humans after consumption of 15 grams of inulin over a 12 h period.^[^ ^67^ ^]^ A trend of lowered isobutyrate suggests that fermentation processes were skewed towards saccharolytic fermentation and that proteolytic fermentation was decreased by inulin intake, which is considered to enhance beneficial metabolic processes, such as insulin sensitivity.^[^ ^68^ ^]^

In order to investigate possible effects of inulin‐induced microbiota changes, either directly or via their metabolites,^[^ ^69^ ^]^ on functional immune responses against pathogens, the participants also received a HB vaccination. The HB vaccine was used because this vaccination protocol requires three doses to induce sufficient protection and is considered to be rather inefficacious.^[^ ^70^ ^]^ It has been shown before in young adults that this protocol is useful for testing the effects of dietary fibers such as inulin on immunity.^[^ ^22^ ^]^ In our study, 81% of the participants were successfully immunized against HB after completing the vaccination protocol, which corresponds with the percentage of responders for this age group in a large Dutch cohort of healthy individuals.^[^ ^71^ ^]^ However, no effect of inulin consumption on the HB titer was observed. Similar results, including changes in microbiota and some immune parameters but not antibody level, were obtained in a population between 45 and 63 years old consuming 8 g day^−1^ of a mixture of short‐ and long‐chain inulin before receiving an influenza vaccine.^[^ ^31^, ^32^ ^]^ Because an increase in HB titer could be observed in young adolescents in our previous study using an identical formulation and consumption regime,^[^ ^22^ ^]^ we believe that the decreased responsiveness of the immune system in elderly is the reason for the absence of an effect in this study. Since ageing impairs many aspects of immunity, including both cellular and humoral responses, identifying the mechanisms and cell types responsible is difficult.

Overall, the described study contributes to the extension of the limited available data on the effects of flexibility and responsiveness of microbiota to food components and associated immunity in elderly. In contrast to adolescents,^[^ ^22^ ^]^ in elderly, the inulin supplement intake did not lead to increased antibody production after HB vaccination, and therefore we can conclude that the humoral immunity boosting effects of inulin are target group dependent. However, chicory long‐chain inulin consumption was found to modify the microbiota composition and microbial fermentation in the elderly in a beneficial direction to the best of our knowledge for the first time with an increased relative abundance of bifidobacteria and other species implicated in health effects. Such effects on microbiota may underlie improvements in bowel habits reported for chicory long‐chain inulin in the elderly.^[^ ^20^ ^]^ Further research is required to determine the impact of this on other physiological health parameters for chronic conditions in elderly, such as general inflammation and mental health issues.

## Conflict of Interest

E.E.V. is an employee of Sensus. The other authors have no financial or commercial conflict of interest to declare.

## Author Contributions

M.B.G.K. and M.E.E. contributed equally to this work. M.B.G.K., M.E.E., S.E.A., E.E.V., M.M.F., and P.d.V. designed the research, M.B.G.K., M.E.E., S.E.A., J.G.M.B., and H.V. performed the experiments and did the analysis, and M.B.G.K., M.E.E., S.E.A., J.G.M.B., H.V., E.E.V., M.M.F., and P.d.V. wrote the paper. All authors read and approved the final manuscript.

## Supporting information

Supporting Information

Supporting Table 2

Supporting Table 3

## Data Availability

The data that support the findings of this study are available from the corresponding author upon reasonable request.
