# Supplementary material for: Flexibility of Gut Microbiota in Ageing Individuals during Dietary Fiber Long‐Chain Inulin Intake
Source: Mol Nutr Food Res. 2021 Jan 25;65(4):2000390. doi: 10.1002/mnfr.202000390 (PMC8138623; doi:10.1002/mnfr.202000390)
Supplement: Supplementary file 1 — Supporting Information [file MNFR-65-2000390-s002.docx]

Molecular Nutrition & Food Research

Supporting information

**Flexibility of gut microbiota in ageing individuals during dietary fiber long-chain inulin intake**

M. B. G. Kiewiet^1†^, M. E. Elderman^1†^, S. El Aidy^2^, J.G.M. Burgerhof^3^, H. Visser^1^, E. E. Vaughan ^4^, M. M. Faas^1^, P. de Vos^1^.

1. Immunoendocrinology, Division of Medical Biology, Department of Pathology and Medical Biology, University of Groningen, University Medical Center Groningen, Hanzeplein 1, 9700 RB Groningen, The Netherlands
2. Department of Molecular Immunology and Microbiology, Groningen Biomolecular Sciences and Biotechnology Institute (GBB), University of Groningen, Nijenborgh 7, 9747 AG, Groningen, The Netherlands
3. Department of Epidemiology, University Medical Center Groningen, University of Groningen, 9713 GZ Groningen, The Netherlands
4. Sensus (Royal Cosun), Oosterlijke Havendijk 15, 4704 RA, Roosendaal, The Netherlands

†These authors contributed equally to this work.

1. **Materials and methods**
   1. **Microbiota analysis and bioinformatics**

In brief, DNA was extracted according to manufacturer’s instructions using the NucleoSpin® Soil Kit (Macherey Nagel, GmbH & Co.). High-throughput sequencing of the V3-V5 hypervariable region of the bacterial 16S rRNA gene was performed on an Illumina MiSeq platform according to the standard protocols with minor adjustments. The V3-V5 region was PCR-amplified using universal primers that contained the adapter overhang nucleotide sequences for forward and reverse index primers. Primers used are: 16S V3-V5 *Fwd* CCTACGGGNGGCWGCAG [1] and 16S V3-V5 *Rev* GGGTTGCGCTCGTTGCGGG [2]. Amplicons were purified using AMPure XP beads (Beckman Coulter, Fullerton, CA, USA) and set up for the index PCR with Nextera XT index primers (Illumina, San Diego, CA, USA). The indexed samples were purified using AMPure XP beads, quantified using the Fragment Analyzer (Agilent, Santa Clare, CA, USA) and equal quantities from each sample were pooled. The resulting pooled library was quantified using the Bioanalyzer 7500 DNA Kit (Agilent) and sequenced using the v3 chemistry (2x300 bp paired-end reads).

- 1. **SCFA analysis**

First, 1.5 mL of deionized water was added to 230-270 mg of feces. The samples were vortexed and allowed to stand at room temperature (RT) for 30 min. After vortexing again, the samples were centrifuged (14.000xg, 10 min, RT) and the supernatant was stored at -20°C until further analysis. The SCFA concentrations were analyzed by gas chromatography (GC). After thawing, samples were vortexed and centrifuged again (14.000xg, 5 min, RT). Then, 500 μL of the fecal samples was mixed with 250 µL 0.15M oxalic acid, after which the sample was again vortexed and centrifuged (14.000xg, 5 min, RT). Samples were analyzed on a Thermo Trace 1300 (Thermo Scientific, Breda, the Netherlands) gas chromotograph equipped with a CP-FFAB CD capillary column (25m x 0.53 mm x 1.00 µm). A constant pressure of 20.0 kPa was applied. Samples were added (0.3 µL) using PTV split injection, with a slit flow of 40 mL/min, at an injection temparture of 200 °C. The temperature profile during GC analysis was as follows: from 100 °C to 180 °C at 8 °C/min, then to 200 °C at 20 °C/min, and held at 200 °C for 5 min. Xcalibur software (Thermo Scientific, Breda, The Netherlands) was used to process the data.

- 1. **Cell staining**

Approximately 1x10^6^ white blood cells were incubated for 10 minutes in FACS buffer (PBS+ 10% FCS (*v*/*v*)) containing 20% (*v*/*v*) normal rat serum (Jackson, Newmarket, UK), followed by incubation in an extracellular antibody mix for 15 minutes. Next, the cells were fixed in FACS lysing solution (BD Biosciences, Breda, the Netherlands) for 30 minutes. Subsequently, samples were washed twice with a permeabilization buffer (eBioscience, Vienna, Austria) after which they were incubated for 10 minutes in permeabilization buffer containing 20% (*v*/*v*) rat serum. Finally, these cells were incubated with an intracellular antibody mix for 15 minutes. Washing was performed in between all incubation steps. The whole procedure was performed on ice and in the dark. All antibodies (see table S1) were diluted in a volume of 50 μl, supplemented to a volume of 50 μl with either FACS buffer (extracellular antibody mix) or permeabilization buffer (intracellular antibody mix).

Cells were analyzed on a FACSVerse flow cytometer (Beckton Dickinson BV, Breda, the Netherlands). Lymphocyte subsets were analyzed using FCS Express software (De Novo, Glendale, CA, USA). Flow cytometry gating strategies are shown in Figure S1. Gating is based on FMOs.

**Supporting tables and figures**

**Table S1.** Flow cytometry antibody panel.

**Table S2.** Complete list of the relative abundance of all detected phyla, family, genus, and species per individual and time point (separate excel file).

**Table S3.** Mean relative abundances of all detected phyla, family, genus, and species of the inulin and treatment groups, with the corresponding fold changes, P-values and FDRs (separate excel file).

**Figure S1.** Flow cytometry gating strategy for T cell populations.

**Figure S2**: Relative abundance of all phyla detected in fecal samples at several time points.

**Table S1.** Flow cytometry antibody panel.

| **Antibody** | **Dilution** | **Company (catalogue number)** |
| --- | --- | --- |
| CD3- Pacific Blue | 25x | BD Pharmingen (558117) |
| CD4-FitC | 50x | Biolegend (357406) |
| CD8-PerCP | 50x | Biolegend (300922) |
| CD45RO-Brilliant Violet 605 | 25x | Biolegend (304238) |
| Tbet-PE/Cy7 | 150x | eBioscience (25-5825-80) |
| FoxP3-APC | 50x | eBioscience (17-4776-42 |


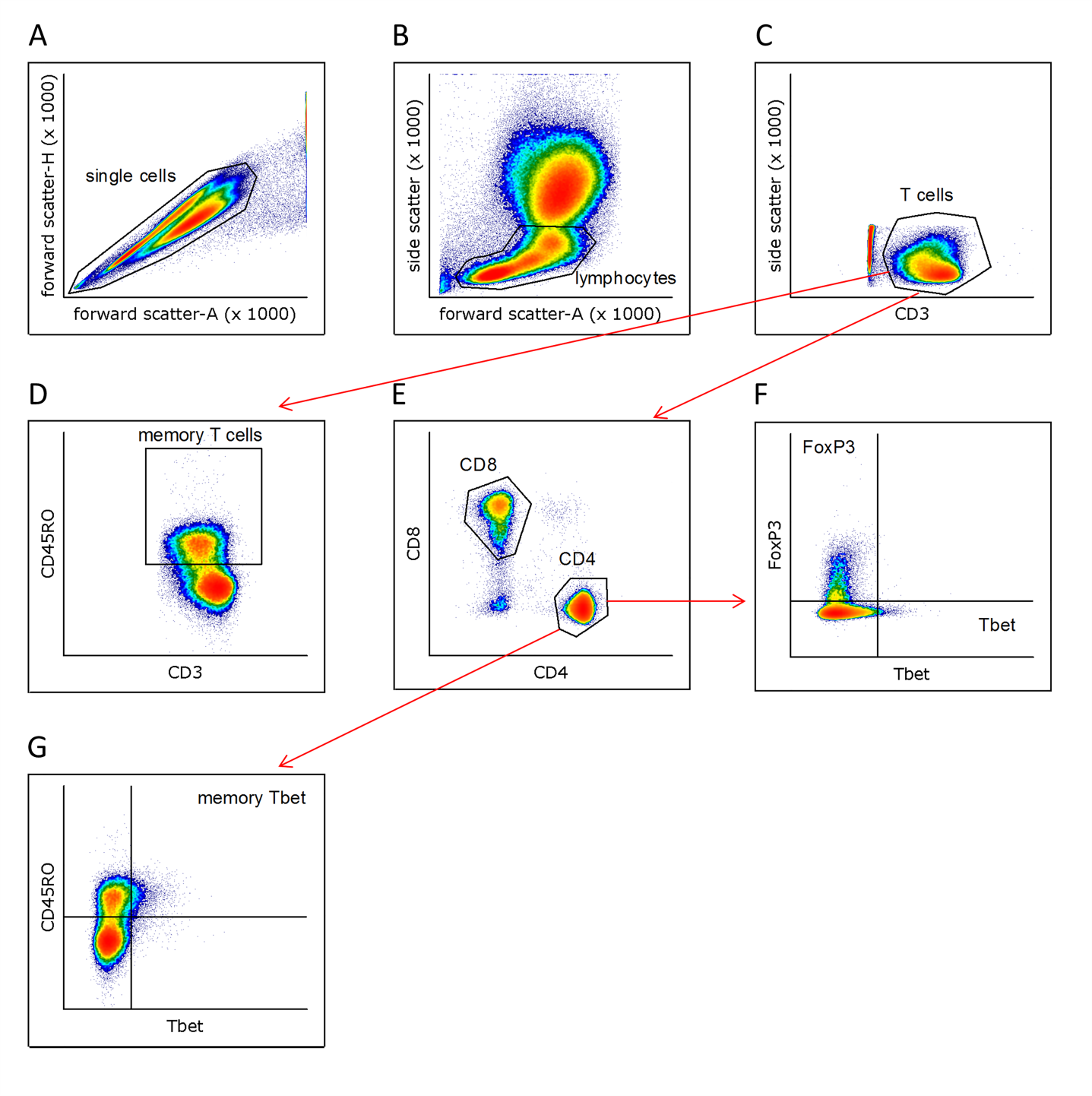
**Figure S1.** Flow cytometry gating strategy for T cell populations. Single cells were selected from the FSC-H vs. FSC-A plot (A), after which the lymphocytes were selected from the SSC-A vs. FSC-A plot (B). CD3 was used as a marker for T cells (C), and within this T cell population CD45RO was used to select memory T cells (D). Next, Thelper (CD4+, E) and cytotoxic T cells (CD8+, E) were gated within the T cell population. Within the Thelper population Th1 (Tbet+, F) and regulatory T cells (FoxP3+, F) were identified. Finally, memory cells within Th1 cells were gated (G).

**Figure S2**: Relative abundance of all phyla detected in fecal samples at several time points. Participants consumed the placebo (control) or inulin daily from day 0 till day 63. Results are shown as relative abundances (A) and were tested for overall treatment and time effects using a LMM on the AUC (B). No significant effect were found.

**References**

[1] Sacchi, C.T., Whitney, A.M., Mayer, L.W., Morey, R., et al., Sequencing of 16S rRNA gene: a rapid tool for identification of Bacillus anthracis. *Emerg. Infect. Dis.* 2002, *8*, 1117–23.

[2] Klindworth, A., Pruesse, E., Schweer, T., Peplies, J., et al., Evaluation of general 16S ribosomal RNA gene PCR primers for classical and next-generation sequencing-based diversity studies. *Nucleic Acids Res.* 2013, *41*, e1.
